# Addition of a sequence from α_2_-antiplasmin transforms human serum albumin into a blood clot component that speeds clot lysis

**DOI:** 10.1186/1472-6750-9-15

**Published:** 2009-03-03

**Authors:** William P Sheffield, Louise J Eltringham-Smith, Sharon Gataiance, Varsha Bhakta

**Affiliations:** 1 Department of Pathology and Molecular Medicine, McMaster University, 1200 Main Street West, Hamilton, Ontario, Canada; 2 Research and Development, Canadian Blood Services, Hamilton, Ontario, Canada

## Abstract

**Background:**

The plasma protein α_2_-antiplasmin (α_2_AP) is cross-linked to fibrin in blood clots by the transglutaminase factor XIIIa, and in that location retards clot lysis. Competition for this effect could be clinically useful in patients with thrombosis. We hypothesized that fusion of N-terminal portions of α_2_-antiplasmin to human serum albumin (HSA) and production of the chimeric proteins in *Pichia pastoris *yeast would produce a stable and effective competitor protein.

**Results:**

Fusion protein α_2_AP(13-42)-HSA was efficiently secreted from transformed yeast and purified preparations contained within a mixed population the full-length intact form, while fusions with longer α_2_AP moieties were inefficiently secreted and/or degraded. The α_2_AP(13-42)-HSA protein, but not recombinant HSA, was cross-linked to both chemical lysine donors and fibrin or fibrinogen by factor XIIIa, although with less rapid kinetics than native α_2_AP. Excess α_2_AP(13-42)-HSA competed with α_2_AP for cross-linking to chemical lysine donors more effectively than a synthetic α_2_AP(13-42) peptide, and reduced the α_2_AP-dependent resistance to fibrinolysis of plasma clots equally effectively as the peptide. Native α_2_AP was found in *in vivo *clots in rabbits to a greater extent than α_2_AP(13-42), however.

**Conclusion:**

In this first report of transfer of transglutamination substrate status from one plasma protein to another, fusion protein α_2_AP(13-42)-HSA was shown to satisfy initial requirements for a long-lasting, well-tolerated competitive inhibitor of α_2_-antiplasmin predicted to act in a clot-localized manner.

## Background

Proteins contain domains, substructures with conserved sequence or structural similarity [1]. The majority of eukaryotic proteins contain multiple domains, ones that have apparently arisen via "domain accretion" resulting from duplication and recombination of the domain-encoding genes [2]. Biotechnological approaches mimic this principle, through the engineering of gene fusions to create expressed proteins, ones combining formerly separate components into a single polypeptide chain. Commonly, the rationale is to slow the clearance from the circulation of injectable protein-based therapeutic agents, or to otherwise stabilize such peptides or proteins. Either immunoglobulins or albumin have been widely used for this purpose [3].

Mammalian albumins are abundant proteins found in blood plasma at mean concentrations of 0.6 – 1.0 mM [4], of 584–585 amino acids in length [5,6]. Unlike most plasma proteins, albumin is not glycosylated, but it nevertheless exhibits a long circulatory half-life [4,7] of 19 days in humans [4]. Recycling of albumin through the major histocompatibility complex-related Fc receptor for IgG (FcRn) has been shown to contribute to its circulatory longevity [8,9]. Productive fusion to albumin has been demonstrated for such diverse proteins as interleukin-2 [10], atrial natriuretic peptide [11], interferon [12], butrylcholinesterase [13] and coagulation factor VII [14]. In the context of improving therapies for thrombosis, our laboratory has explored albumin fusion to improve the pharmacokinetics of small proteins like the thrombin inhibitor hirudin [6,15], the platelet aggregation antagonist barbourin [16,17], and related peptides[18]. Recently, we conceived a new strategy to use albumin fusion to counter thrombosis, by transferring a property of another plasma protein, α_2_-antiplasmin (α_2_AP), to albumin.

α_2_AP is a plasma glycoprotein of the serine proteinase (serpin) superfamily that regulates fibrinolysis, the natural process of blood clot lysis [19]. It binds plasmin using a C-terminal lysine-rich domain and inhibits it by forming a stable, covalent serpin-enzyme complex [20]. It is also cross-linked into fibrin clots, and in that location slows clot lysis by inhibition of plasmin [21]. Chemical [22] or mutational [23] inactivation of the α_2_AP reactive centre loop (RCL) yields a protein that competes with native α_2_AP for cross-linking to fibrin, but which cannot inactivate plasmin. Consequently, clot lysis is accelerated. Activated factor XIII (fXIIIa) mediates the cross-linking of lysines on fibrin and glutamines on α_2_AP [24]. The principal cross-linked residue on α_2_AP is Q14, although other residues can also be modified, and synthetic peptides containing this site are weaker substrates for transglutamination than intact or RCL-inactivated α_2_AP [25].

As a glycoprotein and conformationally sensitive serpin [26], α_2_AP can be difficult to express efficiently in recombinant form. Recombinant human serum albumin (HSA), in contrast, is efficiently expressed at exceptionally high yield in *Pichia pastoris *yeast systems [27]. We hypothesized that N-terminal portions of α_2_AP, when fused to HSA, would confer upon it the ability to be cross-linked to fibrin, and to interfere with α_2_AP-mediated delays in clot lysis. Furthermore, we thought that display on albumin could present Q14 in a more favourable conformation for cross-linking than when it is present in a short peptide. Reasoning that the cross-linking site(s) might require additional motifs from α_2_AP, we compared the production and properties of three overlapping, C-terminally truncated portions of α_2_AP fused to HSA.

## Methods

### DNA Manipulations

Four plasmids were constructed to enable expression of HSA and three α_2_AP-HSA fusion proteins as C-terminally hexahistidine-tagged *Pichia pastoris *expression products. All oligonucleotides were synthesized and all constructs subjected to confirmatory DNA sequencing at MOBIX Lab, a McMaster University core facility. PCR employed Phusion™ high fidelity heat-stable DNA polymerase (NEB). Standard molecular biological protocols for purification of DNA from agarose gels, DNA restriction and ligation, transformation of *E. coli *Top10 (Invitrogen) to ampicillin resistance, and plasmid mini-DNA preparations were employed. First, the HSA DNA was manipulated for expression by PCR amplification, using oligonucleotides ML12007 (5'-CATGGAATTC TTAATGGTGA CGGTGATGGT GTAAGCCTAA GGCAGCTCGA CTTGCAGCAA C-3'), ML12008 (5'-GATCCTCGAG AAAAGAGACG CACACAAGAG TGAGGTTGC-3'), and plasmid pC3HFUS [28] as a template. The resulting 1788 bp amplification product was restricted with XhoI and EcoRI and the inserted between these sites in pPICZ9ssamp [15] to form pPICZ9ssHSAH_6_. PCR products A, B, and C were then generated using as a template the human α_2_AP-encoding plasmid pBAD-H_6_α_2_AP [29]. Codons 13–42 were mobilized using oligonucleotides ML 17226 (5'-ACGTCTCGAGA AAAAGAAACC AGGAGCAGGT GTCCCC-3') and ML 17225 (5'-ACGTGGTAC CGACTCCTGG GGGACTCTTC AG-3') to form product A. Codons 13–73 were obtained using ML 17226 and ML 17227 (5'-AGCTGGTACCC CTGGTGAGCC ACCAGGGAGA AC-3') in product B. Codons 13–109 were amplified using ML 17226 and ML 17228 (5'-AGCTGGTACC CCCAGCACCT TTGCAGCCTC TG-3') to yield product C. The HSA cDNA was then PCR-amplified with oligonucleotides ML 12006 (5'-CATGCGGTAC CACAAGAGTG AGGTTGCTC-3') and ML 12007 (5'-GTTGCTGCAA GTCAGGCTGC CTTAGGCTTA CACCATCACC ATCACCATTA AGAATTCCAT G-3') to produce fragment D. A, B, and C were restricted with XhoI and KpnI and D with KpnI and EcoRI. D was then separately combined with A, B, and C, and inserted between the XhoI and EcoRI sites of pPICZ9ssamp [15], forming plasmids pPICZ9ss-α_2_AP(13-42)-HSAH_6_, pPICZ9ss-α_2_AP(13-73)-HSAH_6_, and pPICZ9ss-α_2_AP(13-109)-HSAH_6_. Each completed, sequence-validated plasmid listed above was used to transform *Pichia pastoris *strain X33 to Zeocin (Invitrogen) resistance as described [18].

### Fusion protein expression, purification, and characterization

HSA fusion proteins were purified from media conditioned by transformed Pichia pastoris cells and induced with 0.5% vol/vol methanol, as previously described for rabbit serum albumin (RSA) fusion proteins [18,30]. Briefly, media was neutralized, separated from cells by centrifugation, treated with protease inhibitors (5 mM benzamidine and 0.1 mM phenylmethylsulfonyl fluoride), and purified using Ni-NTA agarose (Qiagen). Purified proteins were analyzed by SDS-PAGE and immunoblotting using polyclonal goat anti-human α_2_AP antibodies (Affinity Biologicals) and murine monoclonal anti-HSA antibodies (Genway Biotech). They were also characterized by automated Edman degradation at the Advanced Protein Technology Centre of the Hospital for Sick Children, Toronto, Canada. The same facility was the source for synthetic peptides corresponding to α_2_AP(13-42) and to a plasma protein unrelated to this study, heparin cofactor II residues 54–75. The former peptide was shown to contain a single main peak, corresponding to the predicted molecular mass of 3100 Da, by time-of-flight mass spectrometry performed by the manufacturing facility.

### Transglutamination assays using chemical lysine donors

FXIIIa-catalyzed transglutamination of plasma-derived α_2_AP and recombinant α_2_AP-HSA fusion proteins was tested using both artificial (dansyl cadaverine and biotinylated pentylamine) and natural (fibrinogen) lysine donors, in protocols modified from the literature [31]. Purified fXIII, thrombin, and α_2_AP were purchased from Enzyme Research Labs. Human proteins were used in all cases in reactions at 37°C. Using dansyl cadaverine (Sigma) at 0.5 mM, substrate proteins (7.14 μM) were combined with FXIII (1.52 μM) in Tris-buffered saline (20 mM Tris-Cl pH 7.5, 150 mM NaCl) containing 10 mM CaCl_2 _and 0.5 mM dithiothreitol (DTT). Reactions were initiated by the addition of thrombin to 5.0 IU/ml and terminated at specific intervals by addition of disodium EDTA to 10 mM. Transglutamination resulted in protein substrates becoming visible under ultraviolet illumination after SDS-PAGE. Biotinylated pentylamine (BPA; EZ-Link pentylamine-biotin, Pierce) was used under similar conditions with the following exceptions: BPA was substituted for dansyl cadaverine, at 10 mM, FXIII was 0.76 μM, CaCl_2 _was 5 mM and thrombin was 1.0 U/ml. Transglutamination in this case resulted in transfer of a biotin group to substrates. Reactions were visualized on 8% SDS-polyacrylamide gels following transfer to nitrocellulose and blocking with 4.0 mg/ml BSA in TBS/0.2% Tween 20 (TBST), using streptavidin conjugated to horseradish peroxidase (Cedarlane Laboratories) at a 1:5000 dilution. Chemiluminescent substrate cleavage was captured on Biomax XAR scientific imaging film (Kodak). In some experiments band intensity was quantified using UN-Scan-it software (Silk Scientific) and a scanning unit (Canon).

### Transglutamination of fibrin(ogen)

FXIIIa-catalyzed transglutamination of the natural substrate fibrinogen was assessed using SDS-PAGE and immunoblotting of cross-linking reactions. FXIII (100 nM) was quantitatively activated by incubation with 5.0 IU/ml thrombin for 5 minutes at 37°C prior to thrombin inactivation by addition of Phe-Pro-Arg chloromethylketone (FPRck; Calbiochem) to 10 μM. This step eliminated thrombin cleavage of chromogenic substrate S2238 (Diapharma). FXIIIa (at 50 nM final concentration) was then introduced into crosslinking reactions containing 6.0 μM fibrinogen (Sigma), and 1.0 μM substrate proteins in TBS containing 10 mM CaCl_2_. Reactions were stopped by addition of SDS to 1% w/vol, DTT to 50 mM, and urea to 4 M, followed by addition of 0.25 volumes of 4 × SDS loading buffer (125 mM Tris-Cl pH 6.8, 5% SDS, 25% (vol/vol) glycerol, 400 mM DTT, 0.1 mg/ml bromophenol blue). Reactions were electrophoresed on 8% SDS polyacrylamide gels under reducing conditions, and either stained with Coomassie Brilliant Blue or probed with antibodies on immunoblots as described above.

### In vitro clot lysis

Human plasma immunodepleted to < 1% normal α_2_AP levels (ERL) was diluted 1:1 in TBS to final concentrations of 10 mM CaCl_2_, 5 nM thrombin, and 0.125 nM tissue-type plasminogen activator (tPA; single-chain, recombinant tPA, 60 kDa, carrier-free, purchased from Genentech by Dr. Ed Pryzdial, Canadian Blood Services, and University of British Columbia Centre for Blood Research, and kindly donated for our use). Reactions (50 μl) were carried out in microtiter plate wells, and clot formation and lysis was monitored by following changes in turbidity, using an ELx808 plate reader (BioTek Instruments) set to take absorbance readings at 340 nm every 30 seconds for 4 hours. The area under the curve of the resulting profile was measured using data from such experiments transferred to GraphPad Prism 4.0 (GraphPad Software).

### Wessler-type in vivo clot incorporation

The propensity of injected proteins to remain in an *in vivo *clot exposed to flowing blood was determined in anesthetized New Zealand White rabbits using a Wessler-type model, in a protocol modified from the literature [32-34]. Purified human fibrinogen (Sigma) and recombinant proteins were iodinated using the Iodogen method as described by the manufacturer (Pierce), using either sodium ^125^I or ^131^I. Unincorporated radioactivity was removed by exhaustive dialysis versus phosphate-buffered saline. Specific activities of labelling exceeded 1 × 10^9 ^cpm/mg. Anaesthetized rabbits were cannulated via the carotid artery and subjected to a medial incision in the neck to expose the jugular veins. Three ml of whole blood was drawn via the cannula, and anticoagulated with 1/9^th ^volume of 3.8% w/vol sodium citrate. Two centimetre long sections of the right and left jugular veins were isolated, emptied of blood, and blocked off by bulldog clamping. Clotting of the anticoagulated, autologous whole blood was initiated by combining it with warm (37°) human thromboplastin reagent Thromborel S (Dade Behring) in a 1:4 (vol:vol) ratio, supplementing it to 33 × 10^6^cpm/ml ^125^I-fibrinogen and ^131^I-recombinant protein, and the clotting blood (0.15 ml) was re-introduced into the isolated segments. The process was then repeated for the other jugular vein. After 30 minutes of stasis, the clamps were removed, and blood flow restored. One hour later, the jugular veins were opened by longitudinal incision, and the clots were removed, weighed, and γ-counted in a Auto Gamma 5530 Minaxi γ counter (Perkin Elmer), with windows set to discriminate between ^125^I and ^131^I. Values were summed for left and right jugular veins for each animal.

### Statistical analysis

Statistical tests were performed using GraphPad Instat version 3.06 (GraphPad Software). Multiple comparisons used one-way parametric analysis of variation (ANOVA) with Tukey-Kramer post-tests for assignment of group-specific differences, while single comparisons employed Student's t-tests

## Results

### Expression and characterization of fusion proteins

**Figure 1:**
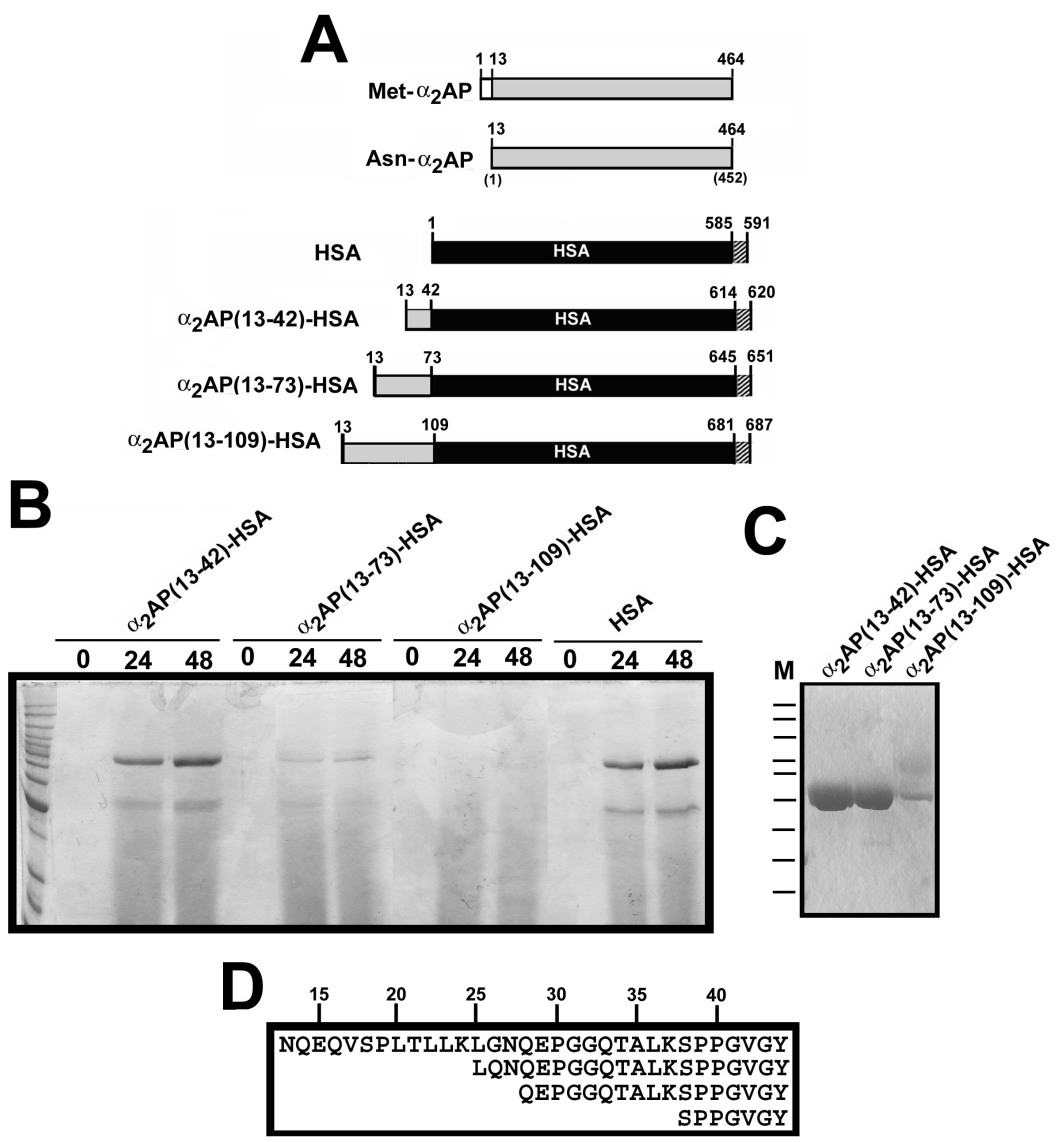
**α_2_AP-HSA fusion protein design, expression, and characterization**. (A) Schematic diagram of proteins. Relevant polypeptides are shown in linear form, with important residues identified above each bar. White bars correspond to the N-terminal dodecapeptide sequence removed from Met-α_2_AP to form Asn-α_2_AP, shown in grey; HSA is shown in black, and C-terminal hexahistidines are shown as stippled bars. (B) Electrophoretic profile of conditioned media samples taken from *P. pastoris *cultures at the times indicated above the lanes, post-induction with methanol. A Coomassie Blue-stained 10% SDS-polyacrylamide gel is shown. Cell lines were transformed with plasmid constructs directing the synthesis of the proteins identified above the horizontal lines. Markers on the leftmost lane of the gel are, in kDa: 160; 140; 120; 100; 90; 80; 70; 60; 50; 40; 30; and 25. (C) A stained gel similar that in panel B is shown, except that 5 μg of the purified proteins identified above the lanes were electrophoresed. M, markers, same as in panel B. (D) N-termini found in purified α_2_AP(13-42)-HSA by amino acid sequencing of the preparation shown in the leftmost lane of panel C. Numbers above the box identify every fifth amino acid residue in α_2_AP(13-42)-HSA.

In human plasma, α_2_AP is found in two forms: a minor form of 464 amino acids commencing with Met (Met-α_2_AP); and a major form of 452 amino acids that arises from processing of Met-α_2_AP at the Pro12-Asn13 bond (Asn-α_2_AP). The latter has been reported to be more efficiently cross-linked to fibrin than the former [35]. We therefore based the 3 α_2_AP-HSA fusion constructs examined in this study on the 13–464 Asn-α_2_AP isoform. As shown in Fig. 1A, we predicted an increase in polypeptide chain length of 30, 61, and 97 amino acids for the three C-terminally hexahisitidinylated fusion proteins versus C-terminally hexahisitidinylated recombinant HSA made in the same *Pichia pastoris *system. This corresponds to molecular masses of 73,127, 76,575, and 80,379 Daltons, respectively, as compared to 67,293 for recombinant HSA. However, as shown in Fig. 1B, comparison of total protein profiles of conditioned media from cultures programmed to produce α_2_AP(13-42)-HSA, α_2_AP(13-73)-HSA, α_2_AP(13-109)-HSA, and recombinant HSA, showed that only in the first and last case did expression of an abundant methanol-inducible protein > 65 kDa ensue. Lesser secretion of the putative α_2_AP(13-73)-HSA and no Coomassie-visible expression of α_2_AP(13-109)-HSA-related species was apparent. Following concentration and purification on nickel-chelate affinity columns, apparently homogeneous preparations of α_2_AP(13-42)-HSA and α_2_AP(13-73)-HSA were obtained (Fig. 1C), although their apparent co-migration suggested either an inability to resolve a 7 kDa difference in the electrophoresis or truncation. In contrast, a heterogeneous preparation of α_2_AP(13-109)-HSA consisting of multiple bands of lesser to equal mobility to the other two proteins was obtained.

Amino acid sequencing of the three preparations shown in Fig. 1C was attempted. No sequence of α_2_AP(13-109)-HSA was obtained. For α_2_AP(13-73)-HSA, a single sequence of VAQTGY, corresponding to residues 70-73 of α_2_AP and a GY dipeptide introduced by DNA manipulations was obtained. This result indicated near-complete proteolysis of the intended α_2_AP(13-73) extension. In contrast, α_2_AP(13-42)-HSA was found to contain a mixture of 4 termini depicted in Fig. 1D. These included the intended full-length sequence, as well as 3 smaller products with N-termini corresponding to L25, Q28, and S38.

### Characterization of recombinant fusion proteins as fXIIIa substrates

**Figure 2:**
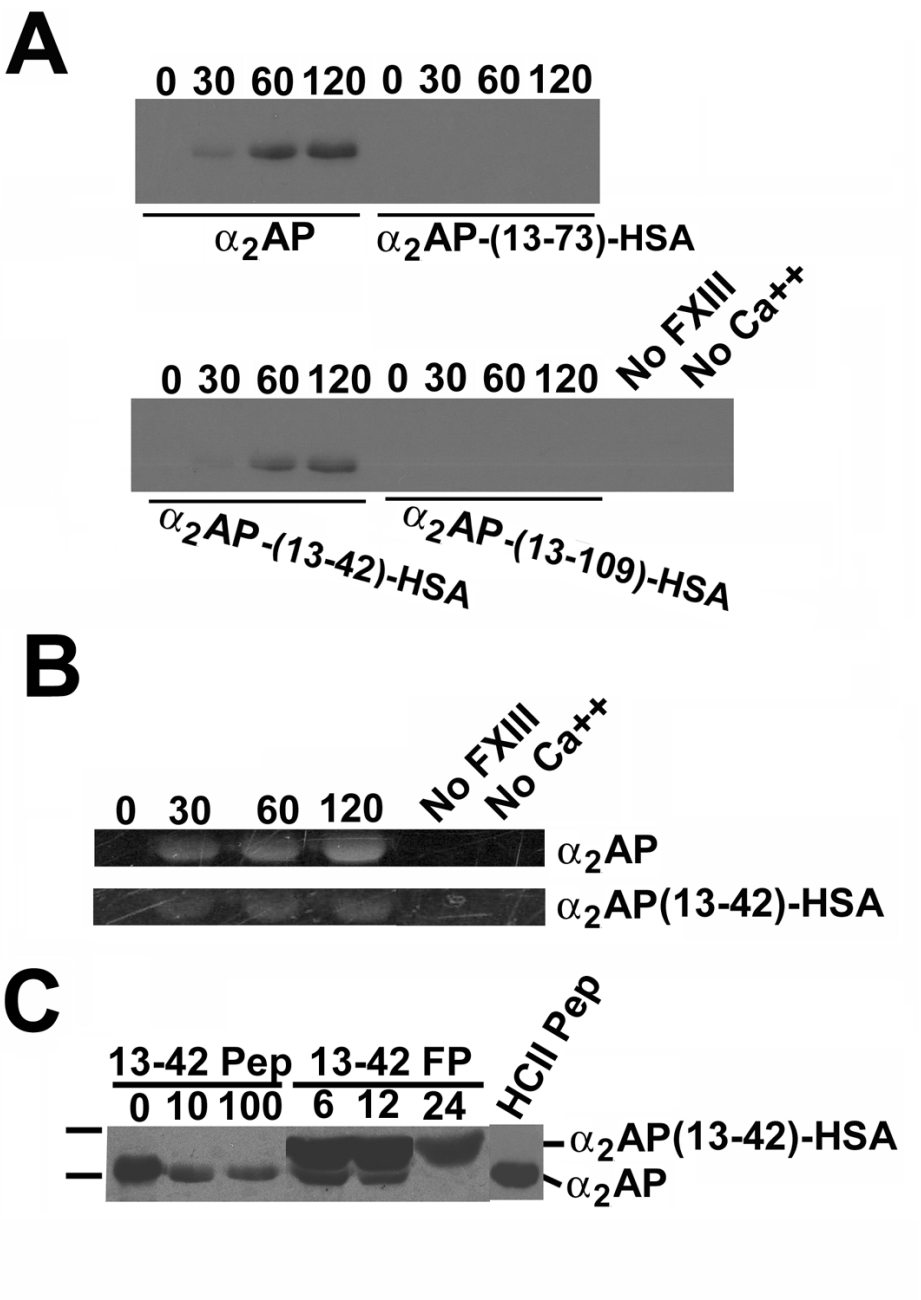
**Factor XIIIa-mediated incorporation of BPA or dansyl cadaverine into α_2_AP and fusion proteins**. (A) Relevant portions of streptavidin blots containing the reaction products of the proteins identified below the panels with 10 mM BPA in the presence of thrombin, FXIII, calcium, and 7.14 μM test proteins, for the times in minutes shown above the lanes, using reaction conditions described in "Methods". Lanes labelled "No FXIII" and "No Ca++" correspond to reactions identical to those shown for α_2_AP at 120 minutes, with the omission of the listed components. (B) Portions of 8% SDS-acrylamide gels visualized by ultraviolet transillumination following fXIIIa-mediated transglutamination of 0.5 mM dansyl cadaverine to the proteins listed the right of the panels. (C) Same as panel A, except that all reactions contained α_2_AP at 1.7 μM, reacted with BPA, thrombin and FXIII in the presence of the concentrations of either α_2_AP(13-42) synthetic peptide (13-42 Pep), α_2_AP(13-42)-HSA (13-42 FP for fusion protein) shown above the lanes. The positions of α_2_AP and α_2_AP(13-42)-HSA are highlighted at right.

The expression and sequencing results predicted that, of the α_2_AP-HSA fusion protein preparations, only α_2_AP(13-42)-HSA had the potential to be a substrate for fXIIIa. This idea was first tested in simple reactions using chemical lysine donors. As shown in Fig. 2A, in the presence of biotinylated pentylamine (BPA), plasma-derived α_2_AP became biotinylated in a fXIIIa- and calcium ion-dependent manner. Similar transglutamination of the biotin-containing lysyl substrate was observed for α_2_AP(13-42)-HSA, with similar kinetics but to a lesser extent. Quantification of band intensities revealed the extent of α_2_AP(13-42)-HSA transglutamination to be 39, 49, and 59%, respectively, of that of α_2_AP at 30, 60, and 90 minutes, respectively. In contrast, α_2_AP(13-73)-HSA and α_2_AP(13-109)-HSA showed no labelling by BPA, in concordance with the electrophoretic and sequencing results. The ability of α_2_AP(13-42-HSA to be transglutaminated to another lysine donor, dansyl cadaverine, was also demonstrated in Fig 2B, although the transfer of dansyl group fluorescence to this protein was less than that observed for α_2_AP.

In the experiment shown in Fig. 2C, BPA-detected transglutamination of α_2_AP was carried out in either the presence of a homogeneous synthetic peptide corresponding to α_2_AP (13-42), or the preparation of α_2_AP(13-42)-HSA shown to contain mixed N-termini by sequencing. Ten- and 100-fold molar excesses of the former reduced but did not eliminate fXIIIa-dependent transglutamination of α_2_AP. An unrelated peptide corresponding to heparin cofactor II residues 54–75 was without effect at 100-fold molar excess. In contrast, a 24-fold molar excess of α_2_AP(13-42)-HSA abrogated transglutamination of plasma-derived α_2_AP, in spite of its heterogeneity at its N-termini.

**Figure 3:**
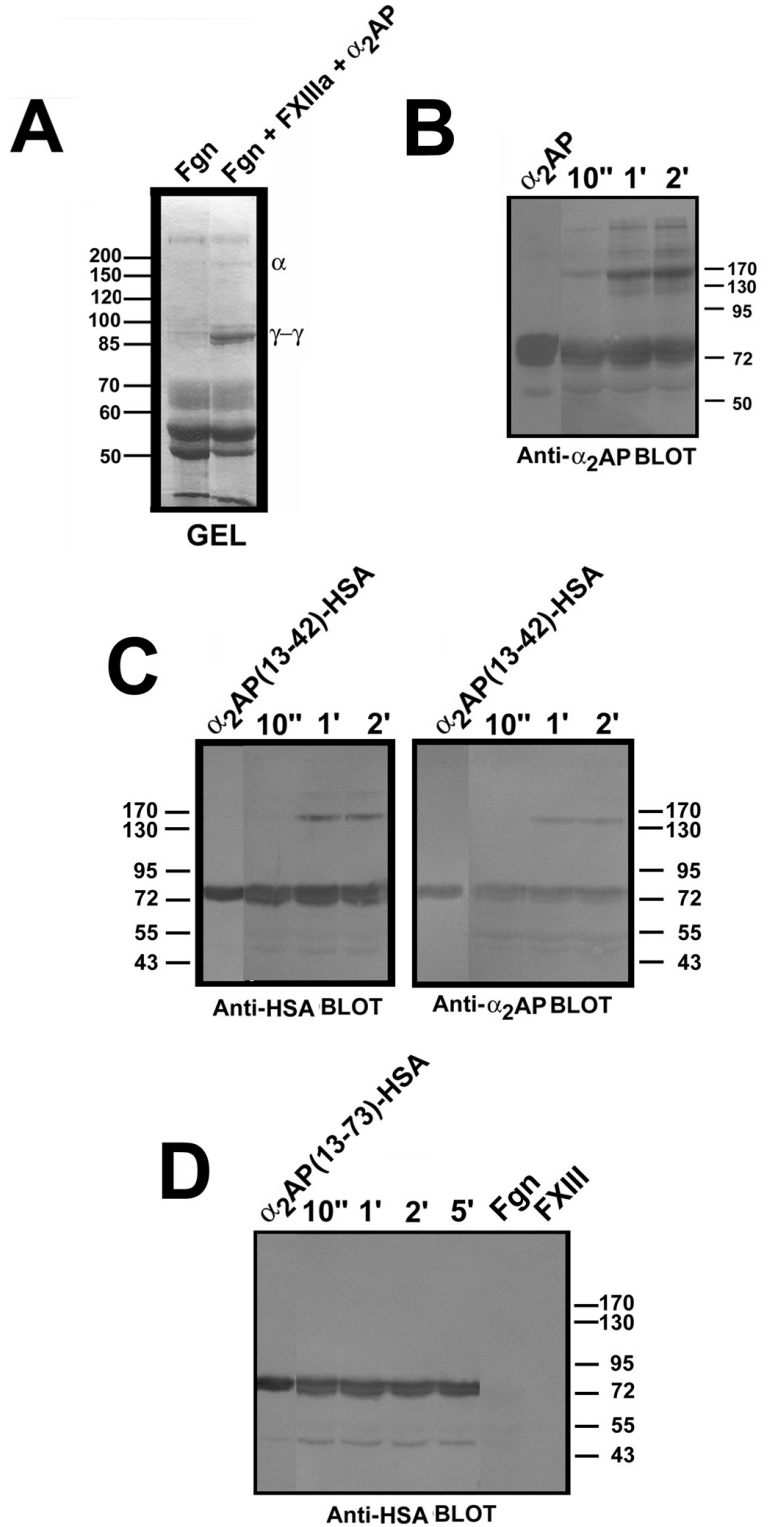
**Cross-linking of α_2_AP and α_2_AP(13-42)-HSA by fXIIIa to fibrinogen**. FXIII was pre-activated to fXIIIa by thrombin, the thrombin inactivated with FPRck, and the fXIIIa then combined with fibrinogen and α_2_AP or α_2_AP(13-42)-HSA or α_2_AP(13-73)-HSA in transglutamination reactions. Reactions were terminated with SDS, DTT, and urea as described in "Methods". (A) depicts a Coomassie Blue-stained SDS-polyacrylamide gel highlighting the cross-linking of fibrinogen into γ-γ dimers (γ-γ) and α-polymers (α). (B) shows an anti-α_2_AP immunoblot of transglutamination reactions containing α_2_AP for the times identified above the lanes; the first lane contains α_2_AP alone. (C), right panel, is identical to B except that α_2_AP(13-42)-HSA was substituted for α_2_AP; (C), left panel, is identical to the right panel except an anti-HSA antibody was used. (D) is identical to (C), left panel, except that α_2_AP(13-73)-HSA was substituted for α_2_AP(13-42)-HSA. The position of molecular mass markers, is shown to the left or right of the panels.

Having demonstrated the potential superiority of α_2_AP(13-42)-HSA over α_2_AP(13-42) as a competitor of α_2_AP cross-linking of a chemical lysine donor, we next asked how effectively it was cross-linked to a natural polypeptide lysine donor, fibrinogen. While fibrin is thought to be the predominant physiological lysine donor for α_2_AP transglutamination, it has been demonstrated that α_2_AP can be cross-linked to circulating fibrinogen [36]. Fibrinogen is a more convenient experimental substrate in that it does not clot, and numerous groups have used it rather than fibrin to examine cross-linking (e.g. [37]). We therefore first examined the ability of α_2_AP(13-42)-HSA to be cross-linked to fibrinogen, utilising sub-physiological concentrations of both fibrinogen and α_2_AP, in order to compare fairly initial rates. As shown in Fig. 3A, fXIIIa catalyzed the formation of readily visualized γ-γ chains (migrating between the 85 and 100 kDa marker positions) and less abundant α polymers (migrating between the 150 kDa and 200 kDa markers) visible by Coomassie Blue staining of SDS-gels. α_2_AP-related protein cross-linking at the concentrations of these proteins that were employed required immunoblotting to detect. As shown in Fig. 3B, higher molecular-weight transglutamination products were detected when plasma-derived α_2_AP was combined with fibrinogen and fXIIIa, whose abundance increased over time and of which a product migrating near the 170 kDa mass marker was arguably the most abundant. Similar results were obtained with α_2_AP(13-42)-HSA, although it was more difficult to visualize this fusion protein than α_2_AP, likely because of the lesser reactivity of the polyclonal anti-α_2_AP with a fusion protein containing only 30 residues of α_2_AP (see Fig. 3C, right panel). When aliquots of the same reactions as those shown in Fig. 3C, right panel, were probed using anti-HSA, an identical pattern was obtained. In contrast, no cross-linked products were obtained when α_2_AP(13-73)-HSA was substituted for α_2_AP(13-42)-HSA in cross-linking reactions (Fig. 3D), even when incubations were prolonged to 5 minutes. Elimination of either fibrinogen or fXIIIa from cross-linking reactions abrogated complex formation (data not shown); antibody specificity, for instance the lack of cross-reaction of anti-HSA monoclonal antibody (Fig. 3D), was as expected. The similar size of the cross-linked products reflected the similar size of glycosylated plasma-derived α_2_AP and α_2_AP(13-42)-HSA; less abundant products of lesser mobility seen in Figs. 3B and 3C resembled those reported by others in similar reactions [31,38].

### Fusion protein α_2_AP(13-42)-HSA competes with α_2_AP in a plasma clot

**Figure 4:**
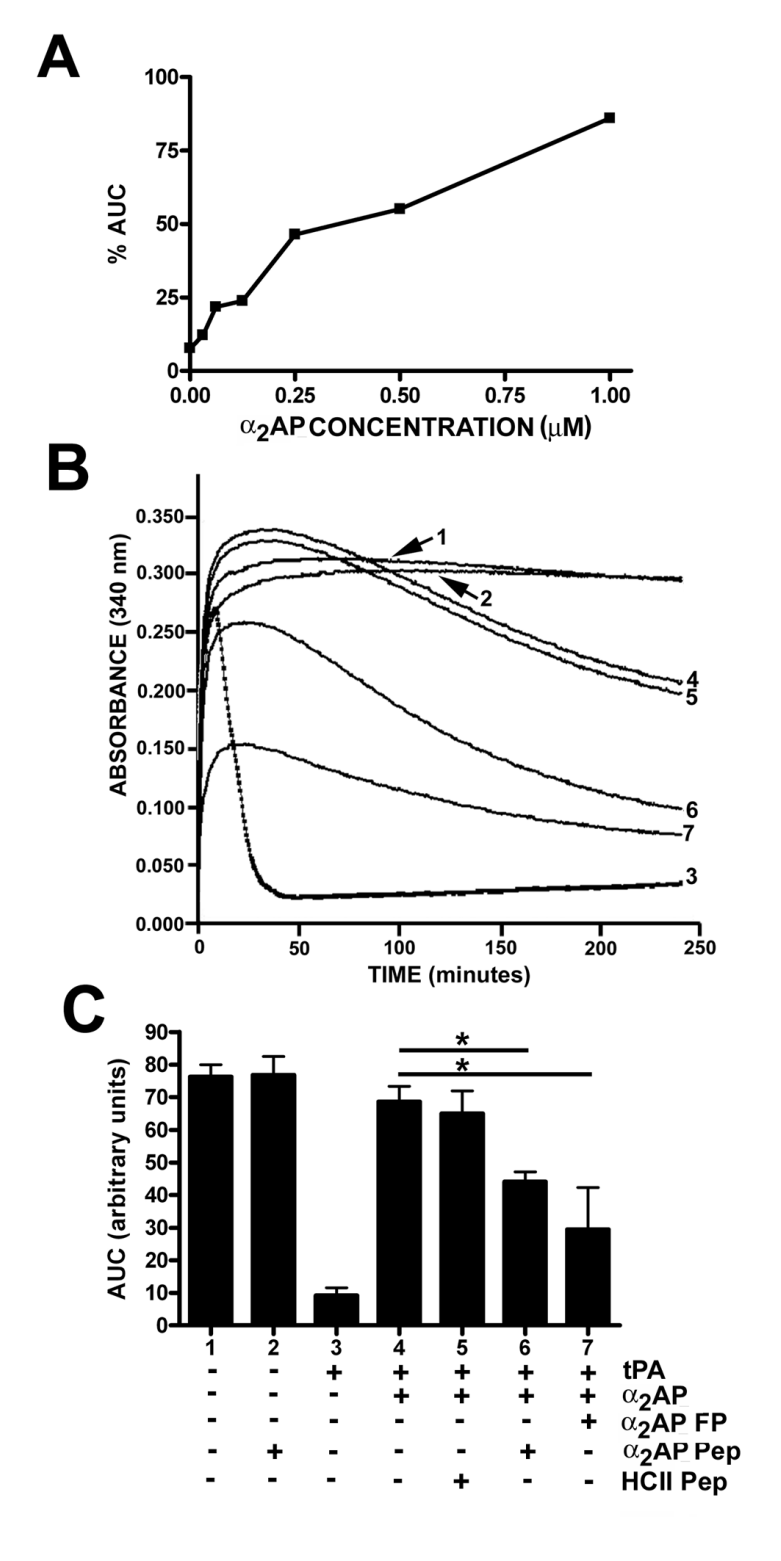
**Effects of α_2_AP and derivatives on plasma clot formation and lysis**. (A) Clot formation and lysis was followed by monitoring turbidity (absorbance at 340 nm) every 30 seconds for 4 hours using a plate reader, of clots formed using diluted α_2_AP-deficient plasma containing both 5 nM thrombin and 0.125 nM tPA, and taking the area under the turbidity versus time curve (AUC). Reactions were supplemented with increasing concentrations of purified plasma-derived α_2_AP, and the AUC relative to that of reactions lacking tPA reported as a percentage. Results of a single experiment are shown. (B) shows turbidity plots for reactions similar to those shown in A, under 7 conditions described in the + or - table in panel C; for instance, (1) shows stable clot formation in the absence of tPA, (3) shows clot formation and rapid lysis in the presence of tPA, (4) shows attenuation of clot lysis in the presence of 1.0 μM α_2_AP and (6) and (7) show competition of the α_2_AP effect by 14 μM α_2_AP(13-42) synthetic peptide (Pep) or α_2_AP(13-42)-HSA fusion protein (FP), respectively. C shows the results of quantification of the experiment shown in B and repeated 4 times (n = 3 to 11 ± SD), under the conditions summarized below the graph, as indicated below the lanes. A control peptide unrelated to α_2_AP corresponding to residues 54–75 of heparin cofactor II was used at 14 μM (HCII Pep). Asterisks indicate significant differences between groups compared between the horizontal lines (p < 0.05).

We next sought to determine if α_2_AP(13-42)-HSA could compete with a physiologically and clinically relevant property of α_2_AP, that of providing resistance to fibrinolysis in clots in which it is cross-linked to fibrin. To ensure the specificity of the effect, we obtained human plasma deficient in α_2_AP. When this anticoagulated plasma was clotted with thrombin, in the presence of calcium ions and low concentrations of tPA, clots formed and then lysed rapidly under the influence of plasmin, whereas if tPA was left out of the reaction, clots were stable for hours (compare turbidity profiles 3 and 1 in Fig. 4B). This difference can be quantified as the area under the turbidity curve. As shown in Fig. 4A, the addition of increasing amounts of purified plasma-derived α_2_AP to this system progressively delayed clot lysis, as has been reported by others, for instance by measuring clot lysis times [22]. Having shown the dependence of this effect on α_2_AP, we added the protein back to its physiological concentration of 1.0 μM, noting that fibrinolysis still occurred, as indicated by the downward slope of turbidity profiles 4 and 5 in Fig. 4B, but at greatly attenuated rates. When a 14-fold molar excess of unrelated peptide HCII 54–75 was added with the α_2_AP, there was no change in the resistance to fibrinolysis. In contrast, use of an identical excess of either α_2_AP(13-42) peptide or α_2_AP(13-42)-HSA fusion protein had a dramatic effect in accelerating clot lysis (compare profiles 6 and 7 to 4 in Fig. 4B). The effect was reproducible and statistically significant (see Fig. 4C), although the greater effect of the fusion protein over the peptide did not reach statistical significance. Similarly, lesser excesses of peptide or fusion protein reduced the mean area under the curve in a dose-dependent manner, but one which did not become significant until 14-fold, the largest amount of fusion protein we could add without re-concentration of our stock solution (data not shown).

### Association of α_2_AP and recombinant proteins with an in vivo clot

**Figure 5:**
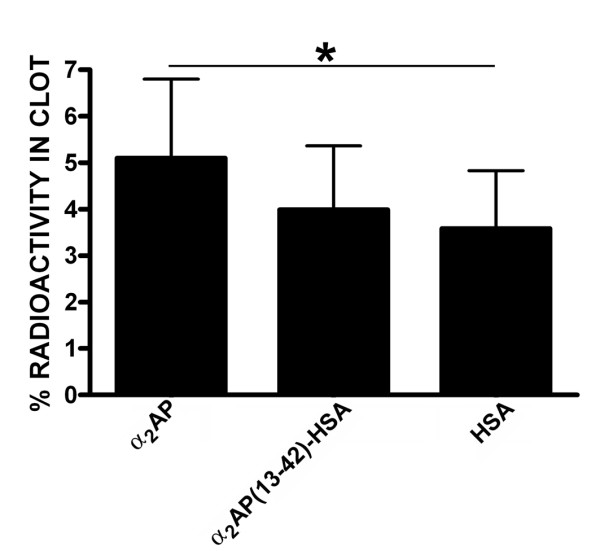
**Localization of radiolabeled proteins in rabbit jugular vein clots in vivo**. The radioactivity remaining in rabbit jugular vein clots allowed to polymerize in clamped-off vessels *in situ *for 30 minutes in the anesthetized animal, then to age with circulation restored for 60 minutes, prior to clot recovery and γ-counting is shown. Clots formed in the presence of ^125^I-fibrinogen and ^131^I-labelled plasma-derived α_2_AP or recombinant α_2_AP(13-42)-HSA or recombinant HSA. Individual data points are the mean of values for both left and right jugular veins, shown as the mean of 6 such means ± SD). Asterisk indicates the only comparison in the group significant (p < 0.05) by paired t-tests.

While the results of both the transglutamination assays and the fibrinolysis resistance experiments suggested successful transfer to albumin of substrate status associated with fusion of α_2_AP(13-42), they also suggested that the fusion protein was a less effective substrate than α_2_AP. We sought to test this conclusion *in vivo *using a Wessler-type model. In this protocol, coagulation is initiated in autologous rabbit blood that is then rapidly introduced into a clamped-off major blood vessel. Clots are allowed to form *in situ*, blood flow is restored, and after aging, the clots are recovered and analysed. ^125^I-fibrinogen was co-injected with a single ^131^I-labelled protein from a group comprised of plasma-derived α_2_AP, recombinant α_2_AP(13-42)-HSA, and recombinant HSA. Figure 5 shows the quantification of the protein-bound radioactivity remaining in the clot at the end of the procedure, normalized to the amount of fibrin(ogen) remaining. The most retained protein was α_2_AP, followed by α_2_AP(13-42)-HSA and HSA. While significantly more α_2_AP was found in the clot than HSA, the amounts of α_2_AP(13-42)-HSA and HSA that were retained were not statistically different. The amount of protein retained in the clot is expected to be the sum of that fraction that is non-covalently trapped and that fraction that is cross-linked. The results support the concept that α_2_AP(13-42)-HSA is a less effective substrate for cross-linking than α_2_AP, not only in vitro but also in vivo, explaining the need for excess fusion protein to compete the effects of the natural antiplasmin.

## Discussion

While it has been known for some time that the principal site of cross-linking of α_2_AP resides in the N-terminal section of the molecule [31,38], short peptides recreating this motif are less efficiently incorporated into fibrin than native α_2_AP. This difference is shown by the finding that 100- to 1000-fold molar excesses of a synthetic peptide corresponding to residues 13–24 of α_2_AP were required to diminish α_2_AP cross-linking to fibrin by 50% [39]. Hypothesizing that anchoring the N-terminal sequence to a protein would conformationally improve its fitness as a fXIIIa substrate, we designed three fusion proteins containing overlapping N-terminal portions of α_2_AP fused to HSA. These segments were rationally selected on the basis of the conserved and aligned secondary structural elements in serpins [40]. Thus, the first selected segment, α_2_AP(13-42), terminates at the beginning of helix A, the second, α_2_AP(13-73), at the beginning of helix B, and the third, α_2_AP (13-109), at the end of combined helices B and C. In addition, choice of the 13–42 segment deliberately avoided residue C43 and the possibility of interfering with the correct pairing of the 17 disulphide bonds in HSA. Finally, Q33 has been reported to be transglutaminated to fibrin or fibrin surrogates, albeit at a 10-fold reduced rate compared to Q14 [31], and it was postulated that its inclusion might increase the reactivity of the chimeric proteins. We had also previously expressed not only small proteins such as hirudin variant 3 (66 amino acids) and barbourin (73 amino acids), as well as smaller barbourin- or RGD-containing derivative peptides as N-terminal fusions to rabbit albumin in the *Pichia pastoris *system employed in this study, without encountering either inefficiency or instability of expression [15,16,18].

In spite of this rational design and previous examples of the soundness of the strategy, the two larger α_2_AP-HSA chimerae were inefficiently expressed in *Pichia pastoris*. While the extreme nature of this expression problem prevented characterization of α_2_AP(13-109)-HSA, α_2_AP(13-73)-HSA was purified and shown to have been proteolyzed at the L69-V70 bond. While we cannot rule out the possibility that this cleavage occurred during the purification process, in spite of the presence of serine protease inhibitors in all solutions, the diminished yield and apparent lack of full-length products in conditioned media samples suggests intracellular proteolysis. In contrast, at least some of α_2_AP(13-42)-HSA protein was produced in full-length form. In two of the three N-terminally truncated chimeric proteins also detected in the purified preparation by N-terminal amino acid sequencing, Q33 was also present and located closer to the N-terminus than in its natural setting, increasing its potential to contribute as a substrate. For these reasons we continued to investigate the α_2_AP(13-42)-HSA, while realising that its functional concentration was probably being underestimated in our assays.

We found that the α_2_AP(13-42)-HSA fusion protein was readily transglutaminated by fXIIIa to both artificial lysine donors like BPA and dansyl cadaverine, and to the natural lysine donor fibrinogen. While the rates of these reactions were noticeably less rapid than that of the fusion protein's natural counterpart, α_2_AP, it was clearly a more effective substrate than its synthetic peptide counterpart, α_2_AP(13-42). This finding was shown through the lower concentrations of fusion protein required to compete α_2_AP cross-linking under at least some conditions.

The specific mechanism by which presentation of α_2_AP(13-42) on the N-terminus of a large protein such as albumin, rather than in untethered free peptide form, would increase its reactivity is not clear. Sugimura *et al*. also reported productive transfer of peptide sequences as fXIIIa substrates to the N-terminus of another protein, in this instance glutathione sulfotransferase [37]. The requirements for efficient cross-linking of sequences by fXIIIa to lysine donors are not well understood, although the suggestion that the reactive glutamine should ideally be positioned in a highly flexible portion of the substrate protein is consistent with our findings [39,41]. It is also likely that the peptide substrates fluctuate between many different conformations in solution, only some of which are optimal for interaction with the transglutaminase, whereas the tethered sequences in the fusion protein context could be constrained in a more optimal conformation.

Although previous reports have highlighted the possibility of using chemically or mutationally modified α_2_AP as antithrombotic agents, by virtue of their ability to reduce α_2_AP-mediated fibrinolytic resistance [22,23], albumin fusion proteins such as α_2_AP(13-42)-HSA, with similar properties, could have significant advantages. α_2_AP has been reported to be inefficiently secreted from *Pichia pastoris *yeast [23], would likely have to be prepared in more expensive mammalian cell culture systems, and has not been investigated clinically or preclinically as a recombinant product in vivo. In contrast, HSA is particularly efficiently expressed in *Pichia pastoris *yeast, a scalable system ideal for economical large-scale pharmaceutical production, and *Pichia*-derived HSA has been shown to have equivalent effects to plasma-derived HSA in patients [42]. Recombinant HSA and HSA fusion proteins exhibit the same or similar plasma longevity to plasma-derived HSA [12,27]. Moreover, there may be immunogenicity advantages to albumin fusion proteins, particularly ones with minimized fusion domains. In a comparison of a 17 amino acid peptide-RSA fusion protein containing the same KGD active antiplatelet sequence found in the 73 amino acid barbourin-RSA fusion protein, the former, but not the latter, elicited antibody formation [18].

In spite of its biotechnological advantages, α_2_AP(13-42)-HSA appears less active in reducing α_2_AP-mediated fibrinolytic resistance in clots than modified α_2_AP proteins, in that an approximately 10-fold molar excess of fusion protein, as opposed to equimolar amounts of modified α_2_AP, are required to halve fibrinolytic resistance in plasma clots in vitro [22,23]. Moreover, α_2_AP(13-42)-HSA demonstrated less ability to become clot-associated in an animal model of thrombosis employed in this study, than native α_2_AP. In this regard, α_2_AP(13-42)-HSA can be viewed as a prototype fusion protein, one that could likely readily be improved in several ways: by mutating residues prone to proteolysis, such as K24 and K37; or by substituting the α_2_AP-derived N-terminal extension with more readily transglutaminated peptides such as those identified by phage display [37]. Such an optimized modified HSA could prove therapeutically useful as an antithrombotic agent, or as an adjunctive drug used in association with thrombolytic therapy.

## Conclusion

An α_2_AP(13-42)-HSA fusion protein was secreted more efficiently by transformed *Pichia pastoris *yeast than chimerae containing longer stretches of α_2_AP. Fusion of the α_2_AP motif changed HSA into a substrate for transglutamination by fXIIIa. The order of reactivity with chemical and physiological lysine donors was α_2_AP > α_2_AP(13-42)-HSA > α_2_AP(13-42) synthetic peptide. The fusion protein reduced α_2_AP-mediated fibrinolytic resistance when present at 14-fold molar excess in plasma clots, but was unable to localize to clots as effectively as α_2_AP in rabbits in vivo. The fusion protein could be the prototype of a well tolerated, long lasting protein drug that counters thrombosis in patients

## Authors' contributions

WPS conceived of the study, secured competitive funding, directed experiments, and wrote the manuscript. LJE-S and SG performed *in vitro *and *in vivo *experiments and refined experimental protocols. VB designed the DNA manipulations and performed some experiments. All authors participated in editing and revising the manuscript.
